# A Kali Phosphoricum Case

**Published:** 1889-02

**Authors:** E. H. Holbrook


### A KALI PHOSPHORICUM CASE.

On evening of December 18th, 1888, I was called to see a child that had been vomiting every little while since the day before. I was informed that she had fallen down two or three steps and commenced vomiting soon after. From the information received, I hardly thought the fall the prime cause of the vomiting, and as it was of a bilious nature I prescribed according to the vomit. The next day she was no better, and the vomited matter was now green and floculent. I now gave *Nat-phos.*$^{cm}$, in water, teaspoonful every hour. When better, every two hours. Was called again that evening. Child was evidently growing worse, and the vomiting increasing in quantity. I was now convinced that the fall was the cause of it, and that the brain had been more severely shocked than was at first supposed. When she falls asleep she lies with the eyes half open; groans a great deal, and is restless; the face is greatly flushed; awakens with a start as if frightened; when awake she complains of pain in the head. She now had a dose of *Kali phos.*$^{cm}$, in water. Soon after taking this she went to sleep and slept for about two hours, when she received another dose, which was about ten o'clock. I ordered, when I found her sleeping quietly after the first dose, that she should not be aroused, but if she awoke or was restless to give it every two hours, but not oftener, and not that often unless necessary. The next morning I saw her about eleven o'clock, and I found her better in every respect—quite bright and sitting up in bed. The mother said she gave the medicine at ten, twelve, three, six, eight, and ten o'clock. There was no more vomiting after giving the remedy until about ten o'clock the next morning, after giving the last dose, and that only a small quantity and greatly changed in character. I now gave *Sac. lac.* every three hours, for the mother to be doing something, and this morning (21st), I found her up and dressed and comparatively well. I found out the child fell farther than was first supposed, and struck the temple probably against the side of the stairs or wall. E. H. HOLBROOK, M. D.

